# LC‐OCT for Cutaneous Lupus Erythematosus: Characterization Across Disease Subtypes and Differentiation From Rosacea

**DOI:** 10.1111/exd.70337

**Published:** 2026-08-03

**Authors:** Daniele Omar Traini, Gerardo Palmisano, Rebecca Ventura, Maria Vittoria Cannizzaro, Clara De Simone, Pierluigi Rizzuti, Viviana Antonella Pacucci, Pier Giacomo Cerasuolo, Silvia Piunno, Cesare Gavotti, Mariarita Vigilante, Alessio Maccari, Giacomo Caldarola, Augusta Ortolan, Mariano Suppa, Alessandro Di Stefani, Maria Antonietta D' Agostino, Ketty Peris

**Affiliations:** ^1^ Dermatology Catholic University of Sacred Heart Rome Italy; ^2^ Dermatology A. Gemelli General Hospital Foundation ‐ IRCCS Rome Italy; ^3^ Rheumatology Catholic University of Sacred Heart Rome Italy; ^4^ Rheumatology A. Gemelli General Hospital Foundation ‐ IRCCS Rome Italy; ^5^ Faculty of Medicine and Surgery Catholic University of Sacred Heart; ^6^ Department of Dermato‐Oncology, Institut Jules Bordet Université Libre de Bruxelles Brussels Belgium; ^7^ Groupe D'Imagerie Cutanée Non Invasive (GICNI) of Société Francais de Dermatologie (SFD), Dermatology Paris France; ^8^ Department of Dermatology, Hôpital Erasme Université Libre de Bruxelles Brussels Belgium

**Keywords:** cutaneous lupus, imaging, LC‐OCT, line field optical coherence tomography, lupus

## Abstract

Cutaneous lupus erythematosus (CLE) encompasses a heterogeneous group of autoimmune skin manifestations that often overlap clinically with other inflammatory dermatoses, particularly rosacea. While histopathology remains the gold standard, its invasiveness limits routine and longitudinal use. Line‐field confocal optical coherence tomography (LC‐OCT) is an emerging non‐invasive imaging modality capable of providing real‐time, near‐histological resolution of skin architecture. In this single‐center cross‐sectional observational study, 45 patients (30 CLE: 16 acute, 5 subacute, 9 chronic; 15 rosacea) underwent LC‐OCT imaging of active lesions. Images were independently evaluated using predefined criteria reflecting histopathological features. LC‐OCT identified key histopathological correlates of CLE in vivo. Interface dermatitis features (including dermoepidermal junction disruption, basal cell vacuolization and band‐like inflammatory infiltrate) were highly prevalent in ACLE and SCLE, while chronic CLE was characterized by epidermal atrophy, hyperkeratosis, fibrosis and adnexal destruction. In contrast, rosacea exhibited a vascular and folliculocentric pattern with frequent peri‐adnexal inflammation and Demodex infestation, without interface changes. Interobserver agreement was substantial to excellent for most criteria. In the ACLE versus rosacea comparison, interface dermatitis features like band‐like DEJ infiltrate, DEJ disruption, basal vacuolization and apoptotic bodies were associated with ACLE. Conversely, rosacea exhibited a vascular‐folliculocentric profile with peri‐infundibular infiltrates and Demodex mites. In conclusion, LC‐OCT enables non‐invasive visualization of disease‐specific microarchitectural patterns in CLE, reflecting both inflammatory and chronic remodelling changes. In this pilot study, it shows potential in differentiating CLE from rosacea and may support diagnosis, staging and treatment monitoring in clinical practice. Larger, multicenter studies are required to confirm the role of LC‐OCT in the non‐invasive diagnosis of CLE.

## Graphical abstract

In this cross‐sectional study of 45 patients, including 30 with cutaneous lupus erythematosus and 15 with rosacea, line‐field confocal optical coherence tomography (LC‐OCT) identified in vivo microarchitectural patterns. Acute and subacute cutaneous lupus were characterized predominantly by interface dermatitis, including dermoepidermal junction disruption, basal vacuolization, band‐like inflammatory infiltrates and apoptotic keratinocytes. Chronic cutaneous lupus also showed hyperkeratosis, epidermal atrophy, fibrosis, follicular plugging and adnexal destruction. Rosacea displayed dilated vessels, peri‐infundibular inflammation and abundance of Demodex mites.

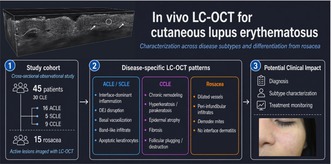

## 1 Introduction

Lupus erythematosus is a chronic autoimmune disease with heterogeneous clinical manifestations, ranging from isolated skin involvement to severe multisystem disease [1]. Cutaneous manifestations are among the most frequent and clinically relevant features of lupus erythematosus and may occur as isolated findings or as part of systemic disease [2]. However, the considerable clinical overlap between cutaneous lupus erythematosus (CLE) and other inflammatory dermatoses frequently complicates differential diagnosis in routine clinical practice [3]. Among common clinical mimickers, rosacea is an important differential diagnosis of facial ACLE because its characteristic centrofacial erythema may be clinically indistinguishable from a ‘malar rash’ [4]. Histopathology remains the gold standard in the diagnostic workup of CLE [5]. Nevertheless, skin biopsy is an invasive procedure, associated with patient discomfort, potential scarring and limited suitability for repeated assessments over time [6]. These limitations highlight the need for reliable, non‐invasive imaging techniques capable of providing in vivo, real‐time information on skin histology and inflammatory activity [7].

Line‐Field Confocal Optical Coherence Tomography (LC‐OCT) is an emerging non‐invasive technique that allows high‐resolution in vivo imaging of the skin with histological resolution up to a depth of about 500 μm, by combining principles of optical coherence tomography (OCT) and reflectance confocal microscope (RCM) [8, 9]. LC‐OCT has been successfully applied to a wide range of dermatological neoplastic, infectious and inflammatory conditions, highlighting its capacity to detect disease‐specific microarchitectural patterns [10, 11, 12, 13]. Previous case reports and preliminary studies support its use in the diagnosis of discoid lupus erythematosus [14, 15], including discoid‐lupus associated cicatricial alopecia [16, 17]. Accordingly, LC‐OCT may enable non‐invasive diagnosis based on characteristic morphologic features [18, 19]. Despite growing interest in the application of LC‐OCT to inflammatory and autoimmune skin diseases [20, 21, 22, 23, 24], its role in the comprehensive evaluation of CLE remains insufficiently characterized, with existing literature largely limited to the discoid subtype.

The aim of this study is to characterize LC‐OCT features across different clinical forms of CLE and to evaluate its potential as a non‐invasive adjunctive tool in the differential diagnosis from rosacea.

## 2 Patients and Methods

We conducted a cross‐sectional single‐center observational study including consecutive patients with CLE evaluated at the Dermatology and Rheumatology Units, Policlinico Universitario Agostino Gemelli (Rome, Italy) between November 2023 and September 2025.

Eligible participants were adults (≥ 18 years) with a diagnosis of CLE and at least one active cutaneous lesion classified as acute CLE (ACLE), subacute CLE (SCLE), or chronic CLE (CCLE) and able to provide written informed consent.

The diagnosis of SCLE and CCLE was established by histopathological examination of lesional skin. In patients with ACLE, the diagnosis was made clinically by board‐certified dermatologists based on compatible lesion morphology and distribution in patients with a previously established diagnosis of systemic lupus erythematosus who fulfilled the 2019 EULAR/ACR classification criteria [25].

Exclusion criteria were: topical treatment applied to the target lesion within 2 weeks prior to imaging, target lesions with superimposed infection, extensive ulceration/erosion, or lesions consisting exclusively of inactive scarring without clinical signs of inflammation; any concomitant inflammatory dermatosis at the site of imaging that could confound image interpretation was also excluded. As a control group, we enrolled consecutive patients with erythematotelangiectatic rosacea, defined by persistent centrofacial erythema with clinically visible telangiectasias, with active centrofacial lesions and no history of lupus.

The study was conducted in accordance with the Declaration of Helsinki and approved by the local ethics committee (Comitato Etico Lazio Area 3; ID: 8276) and is reported in accordance with the STROBE guidelines for observational research. Written informed consent was obtained from all participants.

### 2.1 Clinical Data Collection and Lesion Selection

For each patient, demographic and clinical data, including age, sex, CLE subtype, disease duration, lesion site and current treatments were recorded. Clinical and dermoscopic images were obtained prior to LC‐OCT to allow documentation and side‐by‐side comparison.

One target active lesion was selected for each patient with CLE. In patients with multiple lesions, the target lesion was predefined as the most clinically active lesion based on clinical examination and dermoscopic assessment. An area of adjacent clinically unaffected skin was also imaged as an internal control. In patients with rosacea, a single active centrofacial site was selected based on clinical and dermoscopic evaluation.

### 2.2 LC‐OCT Assessment

LC‐OCT imaging was performed using a commercially available device (DeepLive, DAMAE Medical, Paris, France). For each imaged site, the following acquisitions were obtained: at least three vertical B‐scan images oriented perpendicular to the lesion's longest clinical axis, reaching approximately 400–500 μm depth or the maximum achievable depth, at least one continuous vertical video scan from the epidermis to the superficial dermis, and at least one three‐dimensional image stack (1 × 1 mm) acquired from the most representative area.

All LC‐OCT images were independently evaluated by two dermatologists experienced in LC‐OCT (D.T. and G.P.), blinded to each other's assessments. LC‐OCT features were assessed using a predefined checklist (Table S1) established based on histopathological correlates of CLE [1]. Additionally, we evaluated the presence of Demodex folliculorum, a typical histopathological hallmark of rosacea [26, 27]. Inter‐observer agreement was calculated on these independent ratings. Any discrepancies between readers were subsequently discussed and resolved in a consensus review session, and the consensus scores were used for the final descriptive analyses of feature prevalence. For the CLE vs. rosacea comparison, readers were blinded to previous diagnosis.

### 2.3 Statistical Analysis

LC‐OCT findings were evaluated as counts and percentages of lesions exhibiting each feature within the respective subgroup (ACLE, SCLE, CCLE, rosacea) and in clinically unaffected skin. The inter‐observer agreement was assessed with Cohen's Kappa coefficient (κ) for each criterion. Data management and analysis were performed using R (v4.2) and Microsoft Excel. To specifically address the differential diagnosis of malar rash and rosacea, an exploratory comparison was performed. For each binary LC‐OCT criterion, proportions were compared using Fisher's exact test and effect sizes were reported as odds ratios (ORs) with 95% confidence intervals; when a zero cell occurred, ORs and confidence limits were estimated using a 0.5 continuity correction. To account for multiple comparisons across criteria, *p*‐values were also adjusted using the Benjamini–Hochberg false discovery rate (FDR) procedure.

## 3 Results

A total of 45 patients were included: 30 with CLE and 15 with rosacea. Within the CLE cohort, 16 patients had ACLE, 5 had subacute SCLE and 9 had CCLE. The CLE cohort was predominantly female (25 women, 5 men), with a median age of 38 years (range 19–62). Median disease duration differed across subtypes, being shortest in ACLE (2.5 years), intermediate in SCLE (4 years) and longest in DLE (6 years).

The rosacea cohort had a median age of 44 years (range 23–65) and was similarly female‐predominant (80%). All cases were erythematotelangiectatic, characterized by persistent centrofacial erythema with telangiectasias and none showed phymatous changes. All participants had Fitzpatrick skin phototypes II‐IV.

The ACLE group was relatively homogeneous because all included patients had only active malar lesions. Similarly, the rosacea comparator group was restricted to patients with active centrofacial erythematotelangiectatic rosacea.

The SCLE and CCLE groups were more heterogeneous with respect to lesion location and clinical presentation. Particularly, in the SCLE group, acquisitions were obtained from the back in 3 cases and from the limbs in 2 cases. In the CCLE group, acquisitions were obtained from the face in 3 cases, from the temporal region in 3 cases and from the upper limbs in 2 cases.

### 3.1 LC‐OCT Findings

LC‐OCT detected epidermal abnormalities across CLE subtypes (Table 1). Compact hyperkeratosis with parakeratosis was most frequent in CCLE (8/9, 89%), less common in SCLE (3/5, 60%) and least frequent in ACLE (4/16, 25%). Epidermal atrophy showed a similar distribution, being most prevalent in CCLE (7/9, 78%) compared with SCLE (2/5, 40%) and ACLE (3/16, 19%).

**TABLE 1:** Frequency of LC‐OCT features in acute, subacute and chronic CLE lesions, non‐lesional skin and rosacea.

	Acute lupus (Malar rash lesions) (*n* = 16)	Subacute lupus (*n* = 5)	Chronic lupus (*n* = 9)	Non‐lesional skin (*n* = 30)	Rosacea (*n* = 15)
Epidermis					
Compact hyperkeratosis/parakeratosis	4/16 (25%)	3/5 (60%)	8/9 (89%)	1/30 (3%)	2/15 (13%)
Epidermal atrophy	3/16 (19%)	2/5 (40%)	7/9 (78%)	1/30 (3%)	1/15 (7%)
Acanthosis	0/16 (0%)	0/5 (0%)	0/9 (0%)	0/30 (0%)	3/15 (20%)
Hyporeflective apoptotic keratinocytes	11/16 (69%)	3/5 (60%)	3/9 (33%)	0/30 (0%)	0/15 (0%)
DEJ					
DEJ disruption/indistinctness	13/16 (81%)	4/5 (80%)	5/9 (56%)	0/30 (0%)	0/15 (0%)
Basal cell vacuolization	12/16 (75%)	3/5 (60%)	4/9 (44%)	0/30 (0%)	0/15 (0%)
Band‐like infiltrate (interface dermatitis)	14/16 (88%)	4/5 (80%)	5/9 (56%)	0/30 (0%)	0/15 (0%)
Dermis					
Inflammatory infiltrate	12/16 (75%)	3/5 (60%)	7/9 (78%)	0/30 (0%)	8/15 (53%)
Mucin deposits	4/16 (25%)	2/5 (40%)	3/9 (33%)	2/30 (7%)	0/15 (0%)
Dilated vessels	9/16 (56%)	2/5 (40%)	2/9 (22%)	3/30 (10%)	12/15 (80%)
Fibrotic changes	0/16 (0%)	1/5 (20%)	8/9 (89%)	0/30 (0%)	2/15 (13%)
Adnexal Structures					
Follicular keratotic plugging	1/16 (6%)	1/5 (20%)	8/9 (89%)	0/30 (0%)	4/15 (27%)
Follicular destruction	0/16 (0%)	0/5 (0%)	6/9 (67%)	0/30 (0%)	1/15 (7%)
Peri‐infundibular/periannexal infiltrates	3/16 (19%)	1/5 (20%)	7/9 (78%)	0/30 (0%)	15/15 (100%)
Demodex mites	0/16 (0%)	0/5 (0%)	0/9 (0%)	0/30 (0%)	13/15 (87%)

Hyporeflective apoptotic keratinocytes were more characteristic of ACLE and SCLE, observed in 11/16 ACLE lesions (69%) and 3/5 SCLE lesions (60%), compared with 3/9 CCLE lesions (33%).

Alterations at the DEJ were common across subtypes. An indistinct or disrupted DEJ was seen in 13/16 ACLE lesions (81%), 4/5 SCLE lesions (80%) and 5/9 CCLE lesions (56%), with basal cell vacuolization showing a comparable distribution (ACLE 12/16, 75%; SCLE 3/5, 60%; CCLE 4/9, 44%). A hyperreflective band‐like infiltrate at the DEJ was detected in 14/16 ACLE lesions (88%), 4/5 SCLE lesions (80%) and 5/9 CCLE lesions (56%), and was not observed in non‐lesional skin controls.

Dermal inflammatory infiltrates were frequently observed across CLE subtypes (ACLE 12/16, 75%; SCLE 3/5, 60%; CCLE 7/9, 78%). Dilated dermal vessels were most common in ACLE (9/16, 56%), with lower frequencies in SCLE (2/5, 40%) and CCLE (2/9, 22%). However, dilated vessels were also occasionally noted in non‐lesional skin (3/30, 10%), indicating that this feature, although supportive, was less subtype‐specific than folliculocentric and fibrotic changes. Mucin deposits were identified in all CLE subtypes (ACLE 4/16, 25%; SCLE 2/5, 40%; CCLE 3/9, 33%) and were infrequent in non‐lesional skin (2/30, 7%).

A key discriminator of chronic disease was fibrotic remodelling, identified on LC‐OCT as thickened, parallelized hyperreflective collagen bundles. Fibrotic changes were present in 8/9 CCLE lesions (89%) but were uncommon in SCLE (1/5, 20%) and absent in ACLE (0/16), supporting fibrosis as a defining chronicity marker in this series.

Adnexal involvement varied markedly between subtypes and clustered strongly within CCLE. Follicular keratotic plugging was observed in 8/9 CCLE lesions (89%), compared with 1/16 ACLE lesions (6%) and 1/5 SCLE lesions (20%). Follicular destruction was documented exclusively in CCLE (6/9, 67%), and peri‐infundibular/periadnexal inflammatory infiltrates were likewise more frequent in CCLE (7/9, 78%) than in ACLE (3/16, 19%) and SCLE (1/5, 20%).

### 3.2 Interobserver Agreement

Overall agreement between the two observers was substantial. Complete agreement (κ = 1) was recorded for hyperkeratosis, interface dermatitis, basal cell vacuolization, dilated vessels, fibrotic changes, follicular keratotic plugging, follicular destruction and demodex mites. Agreement was high for epidermal atrophy (κ = 0.86), acanthosis (κ = 0.83) and perifollicular/periannexal inflammatory aggregates (κ = 0.80). The lowest agreement was observed for mucin deposits (κ = 0.53) and hyporeflective apoptotic keratinocytes (κ = 0.46).

### 3.3 Comparison With Rosacea

In the rosacea group (*n* = 15), LC‐OCT showed a predominantly vascular‐folliculocentric pattern, with peri‐infundibular/periadnexal inflammatory infiltrates in all cases (15/15, 100%) and frequent dilated vessels (12/15, 80%). In contrast, features of interface dermatitis were absent (DEJ indistinctness 0/15, basal vacuolization 0/15 and band‐like DEJ infiltrate 0/15). Demodex folliculorum mites were identified in 13/15 (87%) rosacea cases but in none in CLE (0/30).

Exploratory statistical comparison between ACLE malar rash (*n* = 16) and rosacea (*n* = 15) demonstrated a marked separation of LC‐OCT patterns. Interface dermatitis‐related features were strongly associated with ACLE, including a band‐like infiltrate (14/16, 88% vs. 0/15, 0%; Fisher *p* < 0.05), DEJ disruption/indistinctness (13/16, 81% vs. 0/15, 0%; *p* < 0.05), basal cell vacuolization (12/16, 75% vs. 0/15, 0%; *p* < 0.05) and hyporeflective apoptotic keratinocytes (11/16, 69% vs. 0/15, 0%; *p* < 0.05). Conversely, rosacea was characterized by a folliculocentric pattern with peri‐infundibular/periannexal infiltrates (15/15, 100% vs. 3/16, 19%; *p* < 0.05) and frequent visualization of Demodex mites (13/15, 87% vs. 0/16, 0%; *p* = 5.09 × 10^−7^; *p* < 0.05) (Table 2). Other criteria did not reach statistical significance in this ACLE versus rosacea comparison (all q > 0.05) and should be interpreted descriptively.

**TABLE 2:** Frequency of LC‐OCT features in the malar rash, typical of acute cutaneous lupus erythematosus (ACLE) and rosacea.

LC‐OCT criterion	ACLE malar rash (*n* = 16)	Rosacea (*n* = 15)	OR (ACLE vs. Rosacea), 95% CI*	Fisher p	FDR q
Compact hyperkeratosis/parakeratosis	4/16 (25%)	2/15 (13%)	2.167 (0.334–14.06)	0.6539	0.6539
Epidermal atrophy	3/16 (19%)	1/15 (7%)	3.231 (0.297–35.11)	0.5996	0.6424
Acanthosis	0/16 (0%)	3/15 (20%)	0.108 (0.005–2.292)	0.1012	0.1898
Hyporeflective apoptotic keratinocytes	11/16 (69%)	0/15 (0%)	64.82 (3.247–1293.9)	6.77 × 10^−5^	1.69 × 10^−4^
DEJ disruption/indistinctness	13/16 (81%)	0/15 (0%)	119.6 (5.653–2529.1)	3.22 × 10^−6^	1.21 × 10^−5^
Basal cell vacuolization	12/16 (75%)	0/15 (0%)	86.11 (4.222–1756.2)	1.61 × 10^−5^	4.84 × 10^−5^
Band‐like infiltrate (interface dermatitis)	14/16 (88%)	0/15 (0%)	179.8 (7.942–4070.4)	5.09 × 10^−7^	3.82 × 10^−6^
Inflammatory infiltrate	12/16 (75%)	8/15 (53%)	2.625 (0.574–12.00)	0.2734	0.3417
Mucin deposits	4/16 (25%)	0/15 (0%)	11.16 (0.547–227.6)	0.1012	0.1898
Dilated vessels	9/16 (56%)	12/15 (80%)	0.321 (0.065–1.600)	0.2524	0.3417
Fibrotic changes	0/16 (0%)	2/15 (13%)	0.164 (0.007–3.707)	0.2258	0.3387
Follicular keratotic plugging	1/16 (6%)	4/15 (27%)	0.183 (0.018–1.876)	0.1719	0.2865
Follicular destruction	0/16 (0%)	1/15 (7%)	0.293 (0.011–7.765)	0.4839	0.5583
Peri‐infundibular/periadnexal infiltrates	3/16 (19%)	15/15 (100%)	0.008 (0.000–0.177)	3.22 × 10^−6^	1.21 × 10^−5^
Demodex mites	0/16 (0%)	13/15 (87%)	0.006 (0.000–0.127)	5.09 × 10^−7^	3.82 × 10^−6^

## 4 Discussion

**FIGURE 1:**
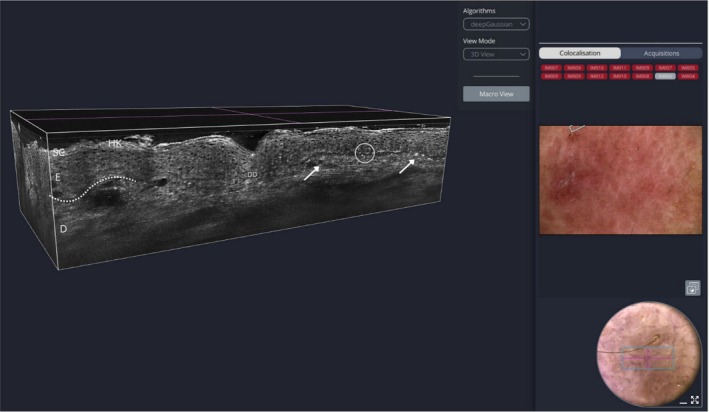
An example of LC‐OCT interface with dermoscopic correlate. The LC‐OCT on a malar rash of a 25‐year‐old woman with cutaneous acute lupus shows a non invasive, in vivo, correlate of histopathology (SC = Stratum Corneum, E = Epidermis, D = dermis, dotted line = Dermal‐epidermal junction). Particularly, are visible hyperkeratosis (HK), basal cell vacuolization (white circle), white hyperreflective inflammatory cells (white arrows) creating a band‐like inflammatory infiltrate.

CLE remains one of the classic ‘great imitators’ in dermatology [2]. Although histopathology is the diagnostic reference standard for CLE, the invasiveness of biopsy, along with patient discomfort and the risk of permanent scarring, particularly in cosmetically sensitive sites such as the face, may limit its use in routine clinical practice. In addition, biopsy is poorly suited for longitudinal assessment. These limitations highlight the need for reliable, non‐invasive techniques capable of reducing the need for biopsies while providing histological‐level information. (Figure 1).

**FIGURE 2:**
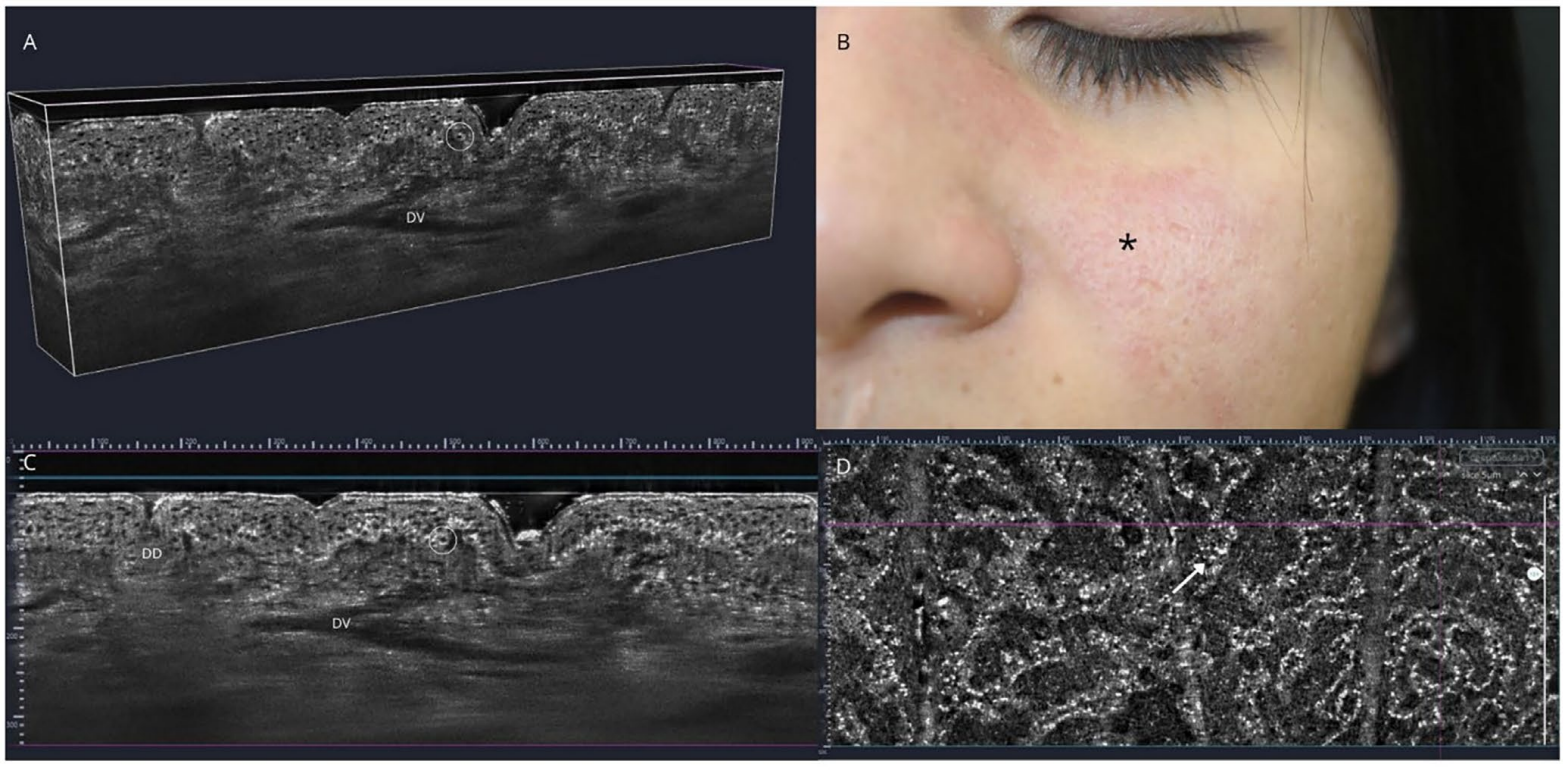
A 38‐year‐old woman with systemic lupus erythematosus and malar rash. Line field confocal optical coherence tomography (LC‐OCT) performed on the malar rash reveals the presence of dilated vessels (DV), basal cell vacuolization (white circle) and interface dermatitis, with presence of inflammatory brights cells (white arrows) at the dermal‐epidermal junction.

**FIGURE 3:**
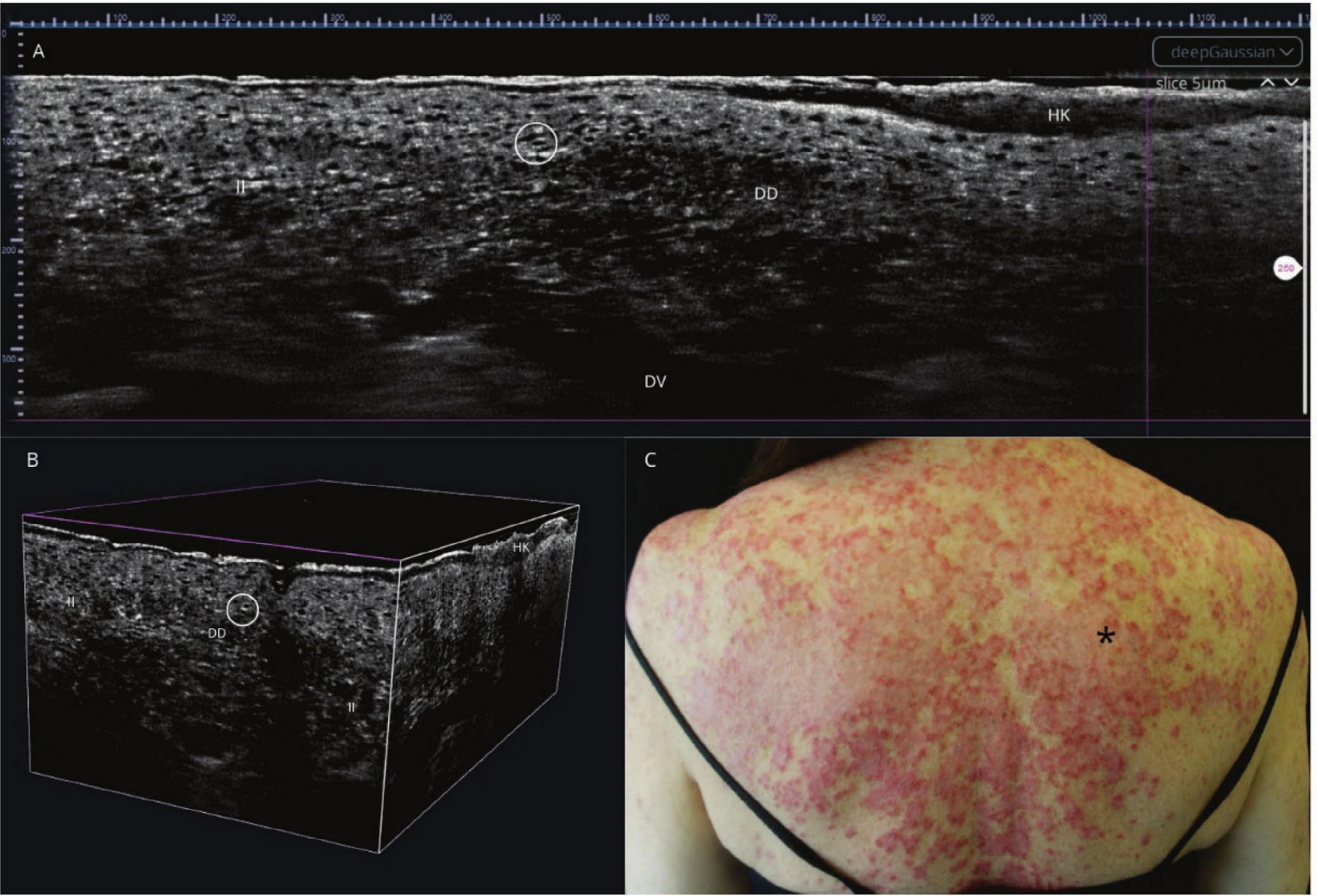
A 62‐year‐old woman with subacute cutaneous lupus. Line field confocal optical coherence tomography, performed on an active lesion (black asterisk) reveals basal cell vacuolization (white circle), dermo‐epidermal junction disruption (DD), hyperkeratosis (HK), dilated vessels (DV) and inflammatory infiltrate (II).

**FIGURE 4:**
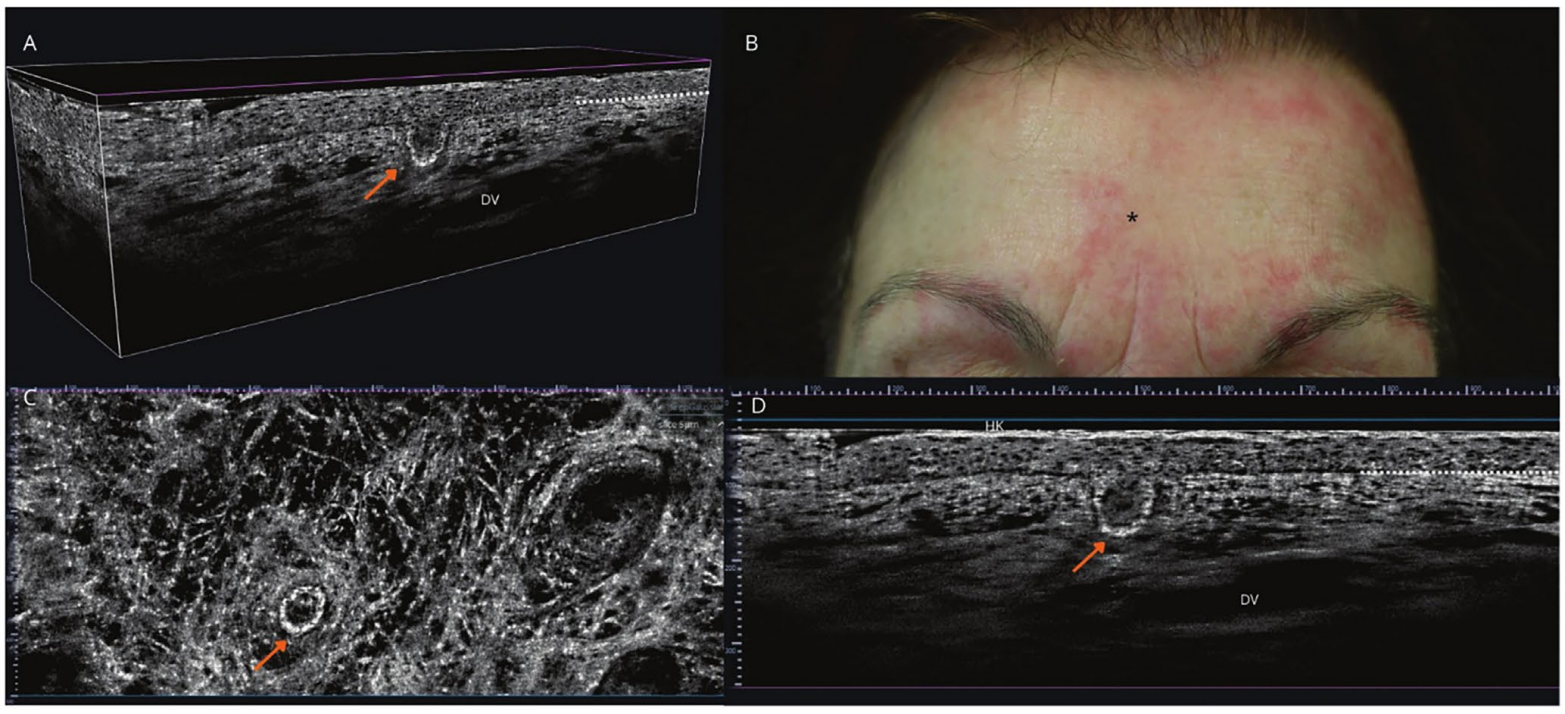
A 57‐year‐old woman with discoid lupus. LC‐OCT shows a perifollicular infiltrate of hyperreflective inflammatory cells (orange arrows), with flattening of dermal‐epidermal junction (dotted line) and dilated vessels (DV).

Our study showed that LC‐OCT was capable of depicting the major histological criteria for the diagnosis of CLE. ACLE (Figure 2) and SCLE (Figure 3) showed high frequencies of interface change (DEJ disruption 81% and 80%; basal vacuolization 75% and 60%; band‐like infiltrate 88% and 80%, respectively) with relatively limited remodelling. Apoptotic keratinocytes were enriched in ACLE/SCLE (69% and 60%) compared with CCLE (33%), consistent with more prominent basal keratinocyte damage in earlier phases [28]. Conversely, CCLE (Figure 4) was characterized predominantly by epidermal and dermal remodelling features (compact hyperkeratosis/parakeratosis 89%, epidermal atrophy 78%, fibrotic changes 89%) and adnexal involvement (follicular plugging 89% and follicular destruction 67%). These findings mirror the scarring process typical of chronic forms [28]. The features we observed in CCLE are in line with those previously described in 15 patients with lupus related alopecia, including lymphocytic interface dermatitis (14/15; 93.3%) and basal cell vacuolization (13/15; 86.7%) and prominent hyperreflective fibres in 14/15 patients (93.3%) [16]. Discoid form of CCLE has been reported to display characteristic histopathologic features, including epidermal atrophy, basal layer vacuolization, focal epidermal‐dermal detachment and a periadnexal inflammatory infiltrate [14]. These observations correspond to the findings of other case series of discoid CCLE [15].

Many features observed, especially inflammatory cells, fibrosis, follicular plugging and follicular destruction, also suggest a role for LC‐OCT in staging and monitoring [29]. Detecting early fibrotic remodelling (Figure S1) or adnexal compromise might encourage earlier therapeutic escalation in patients trending toward scarring disease, while regression of inflammatory signatures (reduced interface banding or decreased inflammatory infiltrate) could provide objective support for treatment response [30].

A clinical scenario in which a non‐invasive technique with histological resolution may be particularly valuable is the differential diagnosis between ACLE and rosacea (Figure S2). Erythematotelangiectatic rosacea typically presents as persistent centrofacial erythema that can be clinically indistinguishable from the malar rash of ACLE [4]. Although ACLE classically spares the nasolabial folds, this sign is not consistently reliable and the distinction may remain uncertain [31]. Diagnostic challenges are further amplified by the fact that both conditions often involve the malar region, a cosmetically sensitive area where biopsy may result in scarring.

In the direct, site‐matched comparison (malar ACLE versus malar rosacea), interface dermatitis signatures (band‐like infiltrate, DEJ disruption, basal vacuolization and apoptotic keratinocytes) were significantly associated with ACLE, whereas folliculocentric inflammation and Demodex visualization were statistically enriched in rosacea after FDR correction. These findings are consistent with known histopathological findings [29]. For example, in a comparative study of 27 rosacea and 30 facial lupus biopsies, follicular plugging, perineural lymphocytic infiltrate, abundant mucin deposition and interface dermatitis were associated with lupus, whereas Demodex infestation and sebaceous hyperplasia were significantly associated with rosacea [31]. While our analyses are hypothesis‐generating, given the relatively small sample, they provide quantitative support for the hypothesis that LC‐OCT can capture, in vivo, the key histopathological discriminators between facial lupus and erythematotelangiectatic rosacea.

Furthermore, interobserver agreement in the substantial‐to‐excellent range in this cohort is encouraging and suggests that LC‐OCT interpretation can be reproducible among trained readers. Agreement being highest for ‘structural’ features such as dermal fibrosis and follicular plugging is also intuitive; these may be the most robust targets for future semi‐quantitative scoring systems.

Limitations of this study are the limited sample size and monocentric design, which may limit generalizability and preclude strong estimates of diagnostic performance. Particularly, given the limited sample size for SCLE and CCLE, the characterization across CLE subtypes remains descriptive.

Moreover, only rosacea (the main differential diagnosis) was used as comparator, but additional CLE mimickers (dermatomyositis, seborrheic dermatitis, allergic/contact dermatitis) were not included. Furthermore, systematic histopathological confirmation was not performed: although histopathology remains the diagnostic gold standard for CLE, systematic biopsy of all imaged lesions was not considered ethically justified, particularly for active facial lesions in cosmetically sensitive areas such as the malar region, when biopsy was not clinically required. ACLE lesions, including those used in the ACLE‐versus‐rosacea comparison, were clinically diagnosed in patients with established SLE rather than histologically confirmed on a lesion‐by‐lesion basis.

Therefore, LC‐OCT findings should be interpreted as in vivo morphologic correlates of established histopathological features rather than as lesion‐by‐lesion histopathologically validated criteria.

Finally, the ACLE‐versus‐rosacea inferential analyses were exploratory, based on a limited sample size and a single rosacea phenotype (erythematotelangiectatic), and therefore require external validation in larger, multicenter cohorts and across broader facial inflammatory differentials.

In clinical practice, LC‐OCT might be useful in several complementary scenarios: increasing diagnostic confidence when clinical morphology is equivocal or overlaps with common mimickers; guiding selection of the most informative biopsy site when histologic confirmation is required (e.g., targeting areas with maximal interface change); and enabling repeated, site‐matched monitoring of inflammatory activity and structural remodelling without scarring, thereby supporting follow‐up and treatment‐response assessment in a minimally invasive manner [19].

## 5 Conclusions

This exploratory study supports LC‐OCT as a potential non‐invasive adjunct for the evaluation of CLE, capable of capturing in vivo correlates of interface dermatitis and chronic remodelling, and of distinguishing CLE from rosacea through a recognizable pattern shift from interface‐dominant to vascular‐folliculocentric inflammation. These findings suggest that LC‐OCT may reduce reliance on invasive sampling and enable repeatable, real‐time assessment in clinically challenging settings. However, larger multicenter studies, including broader differential diagnoses and histopathological correlation when clinically and ethically feasible, are required before LC‐OCT can be incorporated into diagnostic algorithms for CLE.

## Author Contributions

Daniele Omar Traini: conceptualization, methodology, investigation, data curation, visualization, resources, formal analysis, writing – original draft; G. Palmisano: methodology, investigation, data curation, visualization, writing – review and editing; R. Ventura: conceptualization, investigation, data curation, visualization, writing – review and editing; Maria Vittoria Cannizzaro, C. De Simone, P. Rizzuti, V.A. Pacucci, P.G. Cerasuolo, S. Piunno, C. Gavotti, Mariarita Vigilante, A. Maccari, G. Caldarola, A. Ortolan: investigation, data curation, writing – review and editing; Mariano Suppa, A. Di Stefani; Maria Antonietta D'Agostino; Ketty Peris: supervision, resources, methodology, writing – review and editing.

## Funding

The authors have nothing to report.

## Ethics Statement

The procedures followed were in accordance with the ethics standards of the responsible committee on human experimentation (institutional or regional) and with the Helsinki Declaration of 1975, as revised in 1983. The patients in this manuscript have given written informed consent to the publication of their case details.

## Conflicts of Interest

The authors declare no conflicts of interest.

## Supporting information


**Figure S1:** A 56‐year‐old woman with chronic lupus erythematosus (A) showing, on LC‐OCT, dermal fibrotic changes, visible as prominent hyperreflective fibres (yellow arrow) forming a ‘stellate pattern’.
**Figure S2:** A comparison between a case of rosacea (A) and a malar rash in acute cutaneous lupus erythematosus (B). In patient A, multiple mites of demodex species can be observed (red arrow) inside into a single follicule. In the patient B, it can be seen a band‐like infiltrate, with dilated vessels, follicular keratotic plugging (FH), mucin deposit (MD) and basal keratinocyte vacuolization.
**Table S1:** Criteria used for the evaluation of line‐field optical coherence tomography of Lupus.

## Data Availability

The data that support the findings of this study are available on request from the corresponding author. The data are not publicly available due to privacy or ethical restrictions.
