# Supplementary material for: LC‐OCT for Cutaneous Lupus Erythematosus: Characterization Across Disease Subtypes and Differentiation From Rosacea
Source: Exp Dermatol. 2026 Aug 3;35(8):e70337. doi: 10.1111/exd.70337 (PMC13430679; doi:10.1111/exd.70337)
Supplement: Supplementary file 1 — Figure S1: A 56‐year‐old woman with chronic lupus erythematosus (A) showing, on LC‐OCT, dermal fibrotic changes, visible as prominent hyperreflective fibres (yellow arrow) forming a ‘stellate pattern’. Figure S2: A comparison between a case of rosacea (A) and a malar rash in acute cutaneous lupus erythematosus (B). In patient A, multiple mites of demodex species can be observed (red arrow) inside into a single follicule. In the patient B, it can be seen a band‐like infiltrate, with dilated vessels, follicular keratotic plugging (FH), mucin deposit (MD) and basal keratinocyte vacuolization. Table S1: Criteria used for the evaluation of line‐field optical coherence tomography of Lupus. [file EXD-35-e70337-s001.docx]

**Supplementary Table 1 - Criteria used for the evaluation of line-field optical coherence tomography of Lupus**

|  | **Criteria** | **LC-OCT Description** |
| --- | --- | --- |
| **Epidermis** | Compact hyperkeratosis / parakeratosis | Compact hyperkeratosis appears as a thick, homogeneous hyperreflective stratum corneum, whereas parakeratosis shows a less compact, heterogeneous appearance. |
|  | Epidermal atrophy | Thinning of the epidermal layer with reduced rete ridge prominence. |
|  | Acanthosis | Irregular epidermal thickening with uneven layering or distorted epidermal architecture. |
|  | Hyporeflective apoptotic keratinocytes | Discrete, round to ovoid hyporeflective or “signet like” structures within the basal or suprabasal epidermis. |
| **Dermoepidermal Junction (DEJ)** | DEJ disruption / indistinctness | Well-defined, continuous junction in normal skin versus blurred, irregular, or poorly demarcated DEJ in affected areas. |
|  | Basal cell vacuolization | Small hyporeflective clefts or vacuole-like spaces along the basal layer of the epidermis. |
|  | Band-like infiltrate (interface dermatitis) | Continuous or discontinuous hyperreflective band aligned along the DEJ, corresponding to inflammatory infiltrate. |
| **Superficial Dermis** | Inflammatory infiltrate | Hyperreflective cellular aggregates with diffuse pattern |
|  | Mucin deposits | Elongated, ribbon-like hyporeflective structures of dark appearance observed within the superficial and upper dermis, intermingled with ill-defined hyperreflective areas |
|  | Dilated vessels | Enlarged, tubular hyporeflective structures with well-defined walls. |
|  | Fibrotic changes | Thickened, parallelized hyperreflective collagen bundles indicating dermal fibrosis and remodeling. |
| **Adnexal Structures** | Follicular keratotic plugging | Hyperreflective material filling and distending the follicular infundibulum. |
|  | Follicular destruction | Loss or distortion of normal follicular architecture with irregular, poorly defined adnexal structures. |
|  | Peri-infundibular / periannexal infiltrates | Hyperreflective inflammatory aggregates surrounding follicles or other adnexal structures. |
|  |  |  |


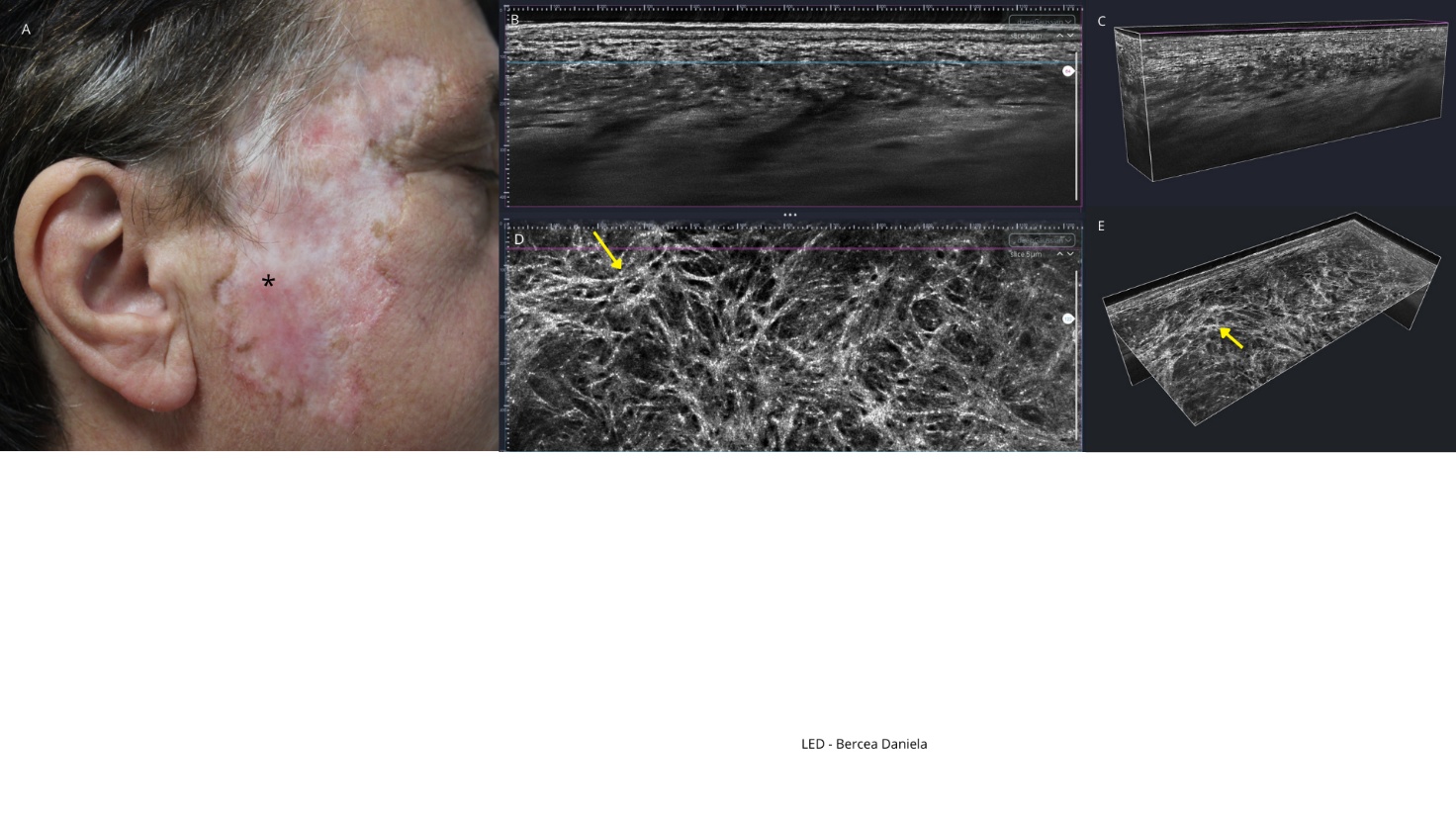


**Supplementary Figure 1**: A 56-year-old woman with chronic lupus erythematosus (A) showing, on LC-OCT, dermal fibrotic changes, visible as prominent hyperreflective fibers (yellow arrow) forming a “stellate pattern”.


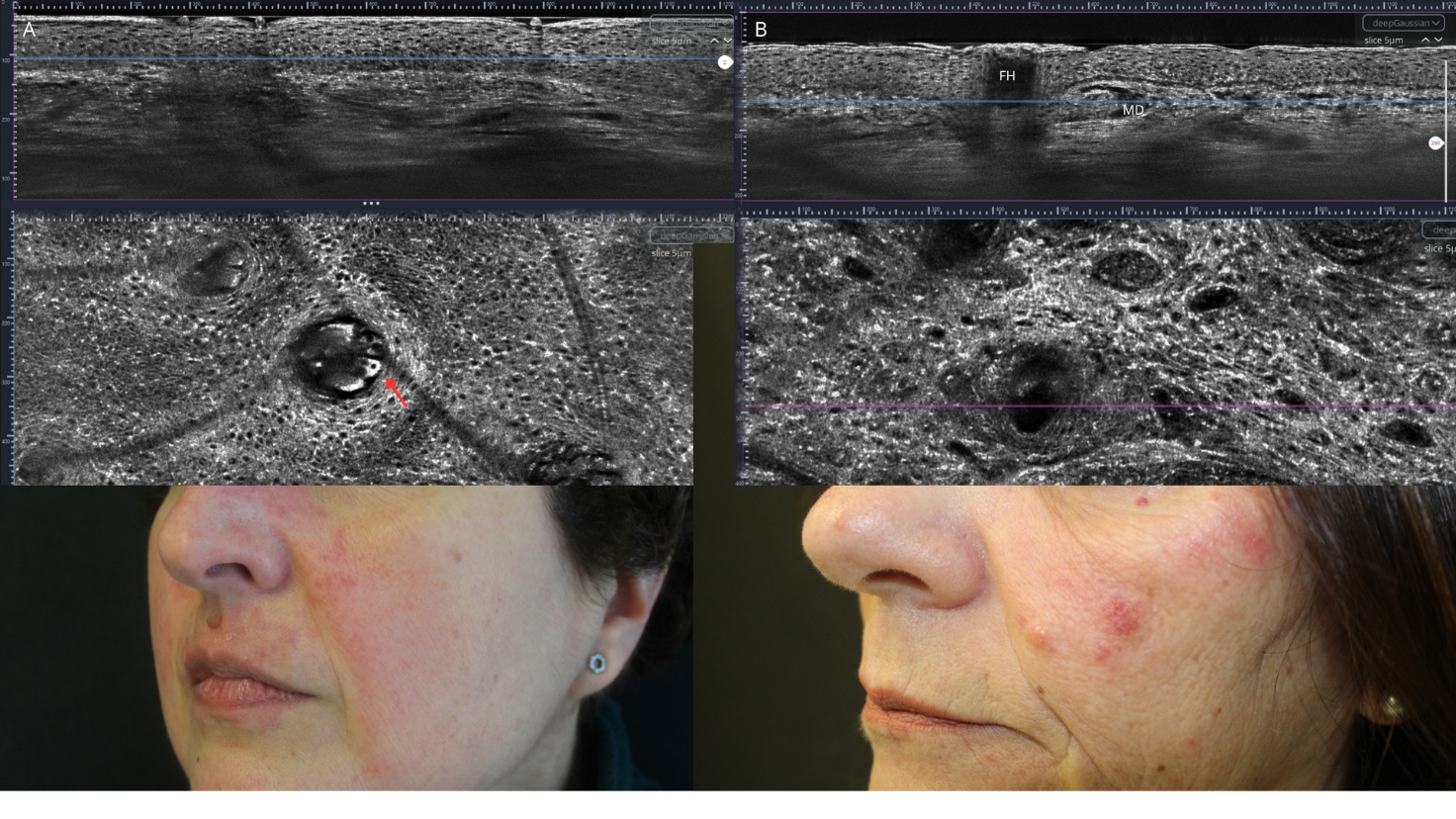


**Supplementary Figure 2:** A comparison between a case of rosacea (A) and a malar rash in acute cutaneous lupus erythematosus (B). In patient A, multiple mites of demodex species can be observed (red arrow) inside into a single follicule. In the patient B, it can be seen a band-like infiltrate, with dilated vessels, follicular keratotic plugging (FH), mucin deposit (MD) and basal keratinocyte vacuolization.
